# Integrating classification and regression learners with bioimpedance methods for estimating weight status in infants and juveniles from the southern Cuba region

**DOI:** 10.1186/s12887-024-04841-9

**Published:** 2024-05-29

**Authors:** Taira Batista Luna, Jose Luis García Bello, Agustín Garzón Carbonell, Ana de la Caridad Román Montoya, Alcibíades Lara Lafargue, Héctor Manuel Camué Ciria, Yohandys A. Zulueta

**Affiliations:** 1 Autonomous University of Santo Domingo (UASD), UASD Nagua Center, Nagua, Dominican Republic; 2 Autonomous University of Santo Domingo (UASD), San Francisco de Macorís Campus, Santo Domingo, Dominican Republic; 3 National Center for Applied Electromagnetism (CNEA), Universidad de Oriente CP 90500, Santiago de Cuba, Cuba; 4 Departamento de Física, Facultad de Ciencias Naturales y Exactas, Universidad de Oriente, Santiago de Cuba, 90500 CP Cuba

**Keywords:** Bioimpedance, Machine learning, Biomedical parameters, Phase angle, Body mass index

## Abstract

**Objective:**

The search for other indicators to assess the weight and nutritional status of individuals is important as it may provide more accurate information and assist in personalized medicine. This work is aimed to develop a machine learning predictions of weigh status derived from bioimpedance measurements and other physical parameters of healthy younger volunteers from Southern Cuba Region.

**Methods:**

A pilot random study at the Pediatrics Hospital was conducted. The volunteers were selected between 2002 and 2008, ranging in age between 2 and 18 years old. In total, 776 female and male volunteers are studied. Along the age and sex in the cohort, volunteers with class I obesity, overweight, underweight and with normal weight are considered. The bioimpedance parameters are obtained by measuring standard tetrapolar whole-body configuration. The bioimpedance analyser is used, collecting fundamental bioelectrical and other parameters of interest. A classification model are performed, followed by a prediction of the body mass index.

**Results:**

The results derived from the classification leaner reveal that the size, body density, phase angle, body mass index, fat-free mass, total body water volume according to Kotler, body surface area, extracellular water according to Kotler and sex largely govern the weight status of this population. In particular, the regression model shows that other bioparameters derived from impedance measurements can be associated with weight status estimation with high accuracy.

**Conclusion:**

The classification and regression predictive models developed in this work are of the great importance to assist the diagnosis of weigh status with high accuracy. These models can be used for prompt weight status evaluation of younger individuals at the Pediatrics Hospital in Santiago de Cuba, Cuba.

## Introduction

Bioimpedance is a technique used to measure the electrical impedance or resistance of biological tissues or fluids. It involves the application of an electrical current, usually through the skin, and the measurement of the resulting voltage [1–4]. Bioimpedance is a non-invasive technique that can provide useful information on various physiological parameters, making it a valuable tool in healthcare and fitness settings. Several physiological parameters can be measured by bioimpedance, including body composition, hydration status, cell membrane integrity, tissue health among other.

The measurement of extracellular and intracellular water (ECW and ICW, respectively) total body water (TBW) is important for estimation of many pathologies [5–9]. Dehydration can be detected by independent measurements of TBW and fat-free mass (FFM), while overhydration may indicate the presence of oedema in cardiac patients or of lymphoedema after a mastectomy [8]. It is well-known that renal patients treated by haemodialysis accumulate fluid between treatments. It is important to evaluate their amount of excess fluid, in order to determine and calibrate the ultrafiltration and also how this fluid loss is distributed between ECW and ICW [7, 8]. An increment of ECM/ICM ratio has been recently associated factor to increased sarcopenia risk in maintenance haemodialysis patients [9]. All of these physiological parameters can be measured by bioimpedance. The development of bioimpedance method surges as an alternative non-invasive methods to get access of these physiological parameters [1–10].

Of particular interest, the health status is associated with weight status of individuals [11–24]. For instance, Obesity is an important risk factor for premature death and the development of a type diabetes, hypertension, heart disease, and chronic kidney disease (CKD) [11–13]. Obesity has increased nearly twice over the past decades [14]. On the other hand, underweight is another risk factor in general health status and has been associated with fractures, lung diseases, atrial fibrillation, cardiovascular diseases and chronic kidney disease [15–24].

According to World Health Organization (WHO), the body mass index (BMI) quantifies the weight status of individuals. There are various classes differentiating the weight status: underweight (BMI < 18.49 Kg/m^2^), normal weight (BMI 18.50–24.99 Kg/m^2^), overweight (BMI 25.0–29.99 Kg/m^2^), class I obesity (BMI 30.0–34.99 Kg/m^2^), class II obesity (BMI 35.0–39.99 Kg/m^2^) and class III obesity (BMI > 40.0 Kg/m^2^) [25]. Besides, this associations vary across global regions and for younger and elder groups [12, 25].

Machine learning is a subfield of artificial intelligence that involves the development of algorithms and statistical models that enable computers to learn from data and make predictions or decisions without being explicitly programmed [26, 27]. In medicine, machine learning has the potential to make more accurate diagnoses, decision making and personalized treatment plans [26, 27].

Despite the development of bioimpedance, there is no direct association between the bioelectrical parameters with weight status. As the WHO system have the lack of variation between global regions and age for estimating the weight status, further studies are required to solve these problems. In this context, the aim of this work is to provide a predictive model, based on classification and regression learner method, as a complementary approach to weigh status evaluation of younger volunteers from the main Pediatrics Hospital of Santiago de Cuba, Cuba.

## Methodology

A pilot random study at the Pediatrics Hospital in South Cuba, specifically in Santiago de Cuba was conducted. This is a public sector hospital specialized in cancer diseases, having the facilities to perform the impedance measurements. The study involved volunteers who were selected between 2002 and 2008 and aged between 2 and 18 years old. In total, 776 female and male volunteers were studied. This research followed the code of ethics, good medical and clinical practices established by the Health General Law of the Ministry of Public Health of Republic of Cuba (Number 41, 13 July 1983 and updated in 2010).

The research was evaluated and approved by the ethics committees and scientific councils of Provincial Blood Bank “Renato Guitart”, Pediatrics Hospital “Conrado Benítez”, Pediatric Hospital “Juan Martinez Maceira” and Oncological Hospital “Antonio María Béguez César”. All relevant national regulations, institutional policies, and the Regional Committees for Medical, Health Research Ethics, and scientific council were in accordance with the tenets of the Helsinki Declaration. Additionally, parents of children and adult participants signed informed consent before starting the study.

The database collected in this study is not publicly available because it is still under study to extract more information that can provide valuable data for a better understanding of the behavior of bioelectrical parameters in healthy and diseased patients. However, the datasets are available from the corresponding author upon reasonable request.

Bioimpedance parameters were obtained by measuring using the standard tetrapolar whole-body configuration. The Bioimpedance analyzer used was the BioScan 98® model (Biológica Tecnología Médica S.L., Barcelona, Spain. URL: http://www.bl-biologica.es). Healthy volunteers participated in a previous fast for at least 3 h, with an empty bladder, and had not exercised or consumed alcohol in the previous 12 h. A 50 kHz frequency was used for the measurements. A disposable pre-gelled Ag/AgCl electrodes model 3 M Red Dot 2560 (3 M, Ontario, Canada) were used.

Trained personnel performed the study in an air-conditioned room set at 23 °C, with a relative humidity of 60–65%, during the morning. The subjects were positioned in a supine posture without clothing or a pillow beneath their heads. Their arms were positioned 30° apart from their chest, while their legs were separated at an angle of 45° without touching each other, on a non-conductive surface. Before electrode placement, the skin was thoroughly cleaned with 70% alcohol. The injector electrodes were placed medial to the dorsal surfaces of the hands and feet, near the third metacarpal and metatarsophalangeal joints. Detector electrodes were placed between the distal epiphyses of the ulna and radius, at the level of the pisiform eminence, as well as at the midpoint between both malleolus respectively. The distance between the injector and detector electrodes was maintained at 5 cm.

To perform a serious AI study, machine learning involves problem formulation, dataset quality analysis, feature selection, and model generalization in the real world [26–28]. The Classification and Regression Learner App implemented in MATLAB code is uses for data analysis and machine learning study [29]. In this study, weight status is considered as the response, and bioelectrical and biological parameters of each volunteer constitute the features. With a large dataset of 776 study cases, a 95% cross-validation for training and 5% for validation are used to avoid the overfitting problem [26–31]. Initially, a feature selection of 20 features is made, followed by a down-selection under the simplest model premise for describing weight status before classification. Various machine learning methods were used for classification and prediction of the body mass index (BMI), and the results presented in the next section are from the best model describing BMI.

## Results and discussion

### Weight status classification model using machine learning techniques

After revising the data, the entire cohort collects 776 infants and juveniles, including 344 females and 432 males. The focus of this study is the weight status as a risk factor for chronic renal failure. Within the cohort, 20 volunteers were class I obese (2.57%), 200 were overweight (25.70%), 277 were underweight (35.69%), and 279 had normal weight (35.95%) based on age and sex. Other weight status classes were not encountered due to the age range. Features considered for training include sex, height, weight, age, resistance (r), capacitive reactance (Xc), phase angle (phase), body surface area (BSA), body mass index (BMI), impedance (Z), total body water volume according to Kotler (TBW_Kotl_), skeletal muscle mass (smmbia), body density (Densbia), fat-free mass (FFM_S_), fat mass (MGC) according to Nhanes, extracellular mass (ECM), intra (ICW_Kotl_) and extracellular water according to Kotler (ECW_Kotl_), basal energetic cost (GBE), and basal metabolic index (IMB).

**Fig. 1:**
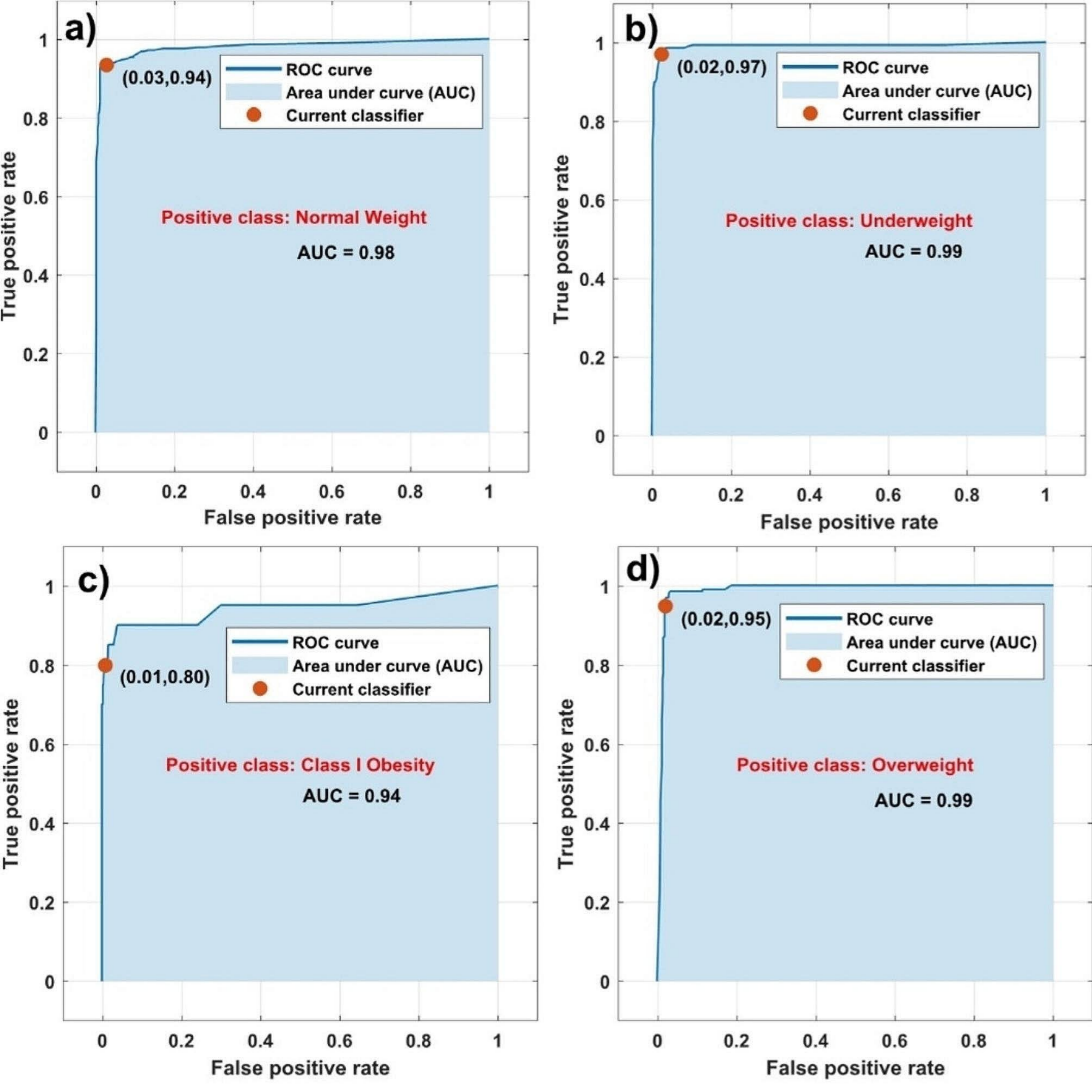
Receiver operating characteristic curve (ROC) for each positive class considered

Results of the training data are depicted in Fig. 1. The best classification model is the bagged trees ensemble with 94.8% accuracy and 40 total misclassification costs. For more accurate predictions, Fig. 1 displays the receiver operating characteristic curve (ROC) for each weight class. The ROC curve provides information regarding the true positive rate versus the false positive rate for a selected trained classifier [32, 33]. The red point highlights the values of the false negative rate (FNR) and the true positive rate (TPR) for the classifier. Additionally, the area under the curve (AUC) number is an indicator of the accuracy of the classifier model [32, 33]. A larger AUC value indicates better classifier accuracy and predictions [32, 33]. The classification model made good predictions in all positive classes considered in this study with AUC > 0.93. From the red points highlighted in the figure, it can be seen that the classification model assigns 3.00% of the false positive rate observations incorrectly to the positive normal weight class with an AUC of 0.94. Analogously, 2.00% of the false positive rate observations are wrongly assigned to the positive underweight and overweight classes with AUC = 0.99. Despite a relatively good AUC value in the case of class I obesity (Fig. 1c), there is a difference between the false positive and true positive rate observations, resulting in less accuracy compared to the other weight status classes. This finding is attributed to only 2.60% of the dataset having class I obesity, resulting in worse performance of the model compared to the other weight status classes.

**Fig. 2:**
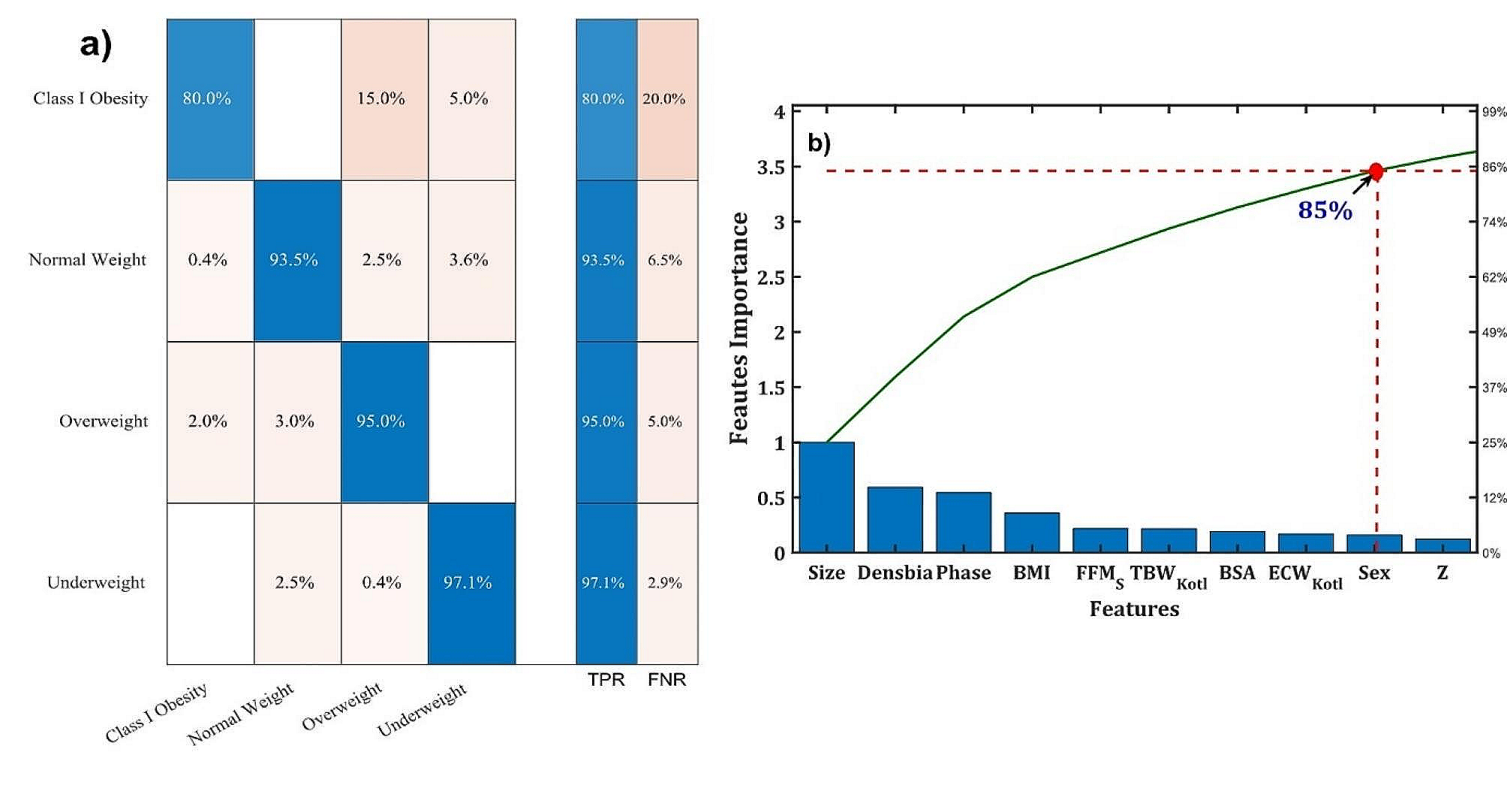
**(a)** Correlation matrix and **(b)** Pareto plots of the weight status classification model

Feature importance separates the main features with direct influence of the model prediction, avoiding the common over fitting problems [26–31]. Figure 2 displays the feature importance represented in a Pareto plot. As it is shown in the Pareto plot, the most influencer features are the size, body density (Densbia), phase angle (Phase), body mass index (BMI), fat-free mass (FFM_S_), total body water volume according to Kotler (TBW_Kotl_), body surface area (BSA), extracellular water according to Kotler (ECW_Kotl_), sex and -with minor relevance- the impedance (Z).

After applying the Pareto rule or the law of vital few and trivial many, which assumes that 85% of the importance of a classification prediction is derived from a few vital features, one can identify the most significant characteristics to include in future regression-classification models. The selected features, in order of importance, are Size, Densbia, Phase, BMI, FFM_S_, TBW_Kotl_, BSA, ECW_Kotl_, and Sex. Figure 2 (red lines) illustrates the Pareto plot used to determine the intercept between the 85% horizontal line and the green curve describing the importance, with the corresponding abscissa feature representing the last characteristic considered for future regression models. Overall, the size, body density, phase angle, and body mass index are the primary determinants of weight condition, as demonstrated by Fig. 2. These characteristics are used in the next section to perform a predictive regression model of weight status.

The results derived from the features importance analysis suggest that other bioimpedance-derived characteristics can be used alternatively for weight status predictions. Specifically, the phase angle and body density have a greater contribution to determining weight status than the common body mass index. Although some reports have described the failure of bioimpedance spectroscopy to determine weight status, other studies have suggested that bioimpedance measurements are not inferior to BMI as a predictor of overall adiposity in a general population [34, 35]. Amani and coworkers have stated that BIA and BMI methods can similarly detect normal and obese women but are less accurate in determining underweight female subjects [34]. A pilot study of 200 Taiwanese women with breast cancer, which combined BIA and BMI, revealed the underestimation of the World Health Organization (WHO) criterion to state the cut-off of younger women with breast cancer [36]. The results suggest that other parameters, namely the phase and body density, can be used to support the diagnosis of weight status.

### Developing a regression-based machine learning model for BMI prediction

**Fig. 3:**
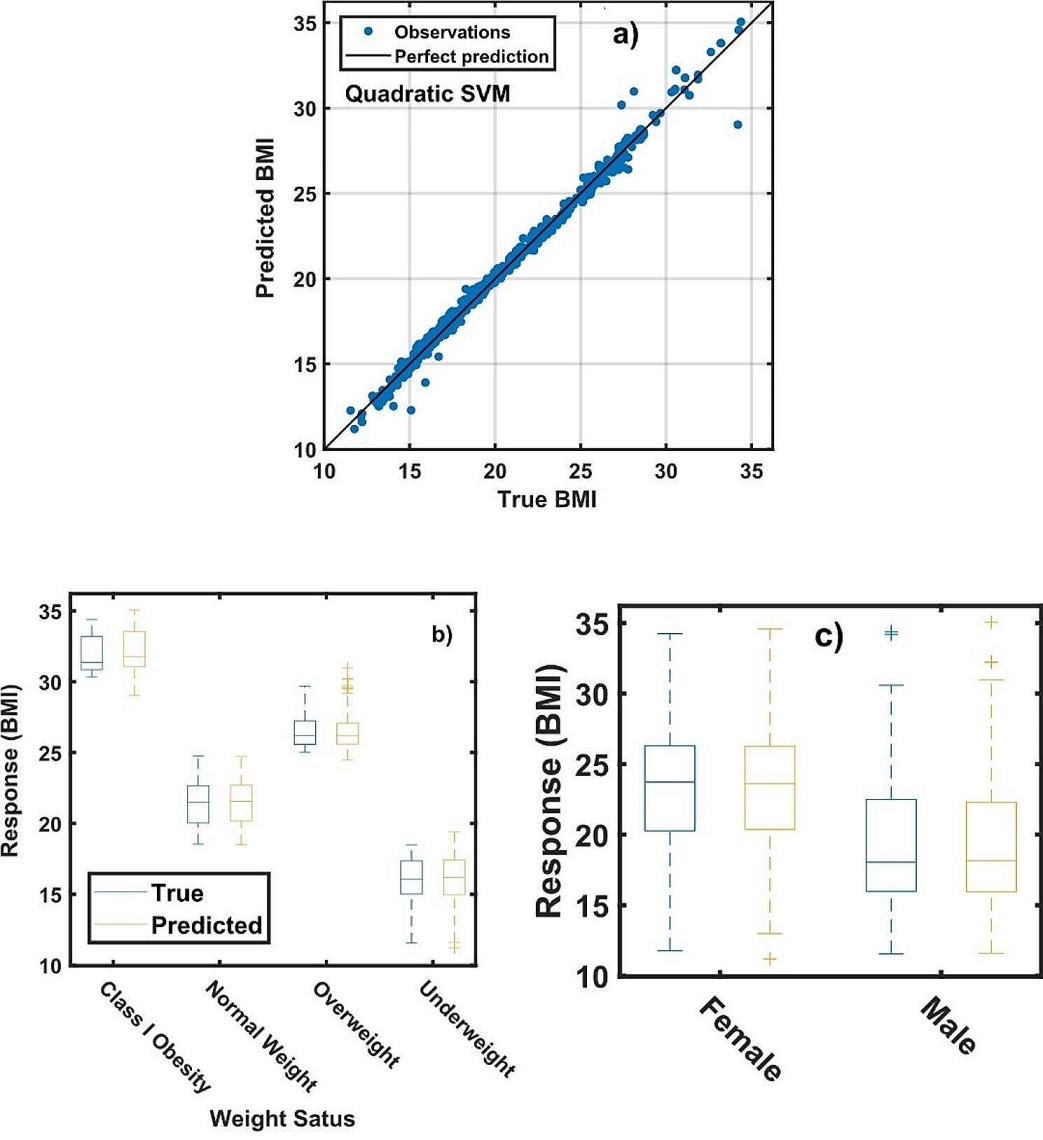
**(a)** Response vs. predicted plot of body mass index (BMI), **(b)** predicted and original BMI data

With the selected features in Sect. 3.1, the next step deals with the machine learning predictions. The quadratic support vector machine (SVM) model is selected for predicting the BMI, which is actually used to identify the weight status. The data is divided in two parts, 95% for training and 5% for cross validation. Figure 3a shows the response versus predicted plot of the body mass index. The accuracy is evaluated by the RMSE = 0.475, MSE = 0.181, R^2^ = 0.999 and MAE = 0.265, indicating a good generalizability and high relevance of the identified features toward explaining relative trends of the weight status. All points lie near of the control straight line, revealing that the chosen model replicates the observations.

Figure 3b displays the response behaviour of BMI of the predicted and original weight status. For the class I obesity, the median value of the original data is 31.36 kg/m^2^, while the predicted value is 31.77 kg/m^2^. Analogously, a median BMI of 21.48, 26.20, and 16.06 kg/m^2^ are observed for normal weight, overweight and underweight status, respectively, are found, and the predicted median BMI are close to the observable values. Underweight status has been associated with an increased risk of end-stage kidney disease [24]. Kim and co-workers [24] studied 26,406 participants diagnosed with end-stage kidney disease. After fully adjusting for other potential predictors, the moderate to severe underweight group (< 17 kg/m^2^) had a significantly higher risk of end-stage kidney disease than that of the normal weight group [24]. On the other hand, obesity also is associated with chronic kidney disease [11, 12]. In this sense, the model can be used to make predictions of weight status. From Fig. 3c, girls have larger BMI than boys, girls habitually accumulate more adipose tissue than boys, and the predictive model derived in the present study reproduces this fact.

### Developing a classification model for determining weight status in an adolescent cohort

Controlling the weight status of adolescent will improve their health during to adulthood [37–41]. Several large longitudinal studies have shown that obese and overweight children who achieve a healthy weight later in life have similar cardiovascular risks as individuals who were never obese. Other diseases such as metabolic syndrome, left ventricular hypertrophy and geometry in hypertensive children have been related to the weight status in adolescents [37–41]. In this section a predictive model of the weight status taking from the data the adolescent cohort ranging between 12 and 18 years old are performed. The adolescent cohort (selected from the 2 to 18 years old cohort) contain 548 individuals, among them 35 male and 514 female, 252 normal weight, 177 overweight, 103 underweight and 16 class I obesity volunteers. The same feature selection and conditions are adopted from Sect. 3.1, but in this case the BMI was not included as a characteristic for making weight status predictions. The best classification model is the bagged threes ensemble with 97.10% of accuracy and 16 total misclassification cost. Note that the model has better accuracy as compared with the entire age cohort discussed in Sect. 3.1.

**Fig. 4:**
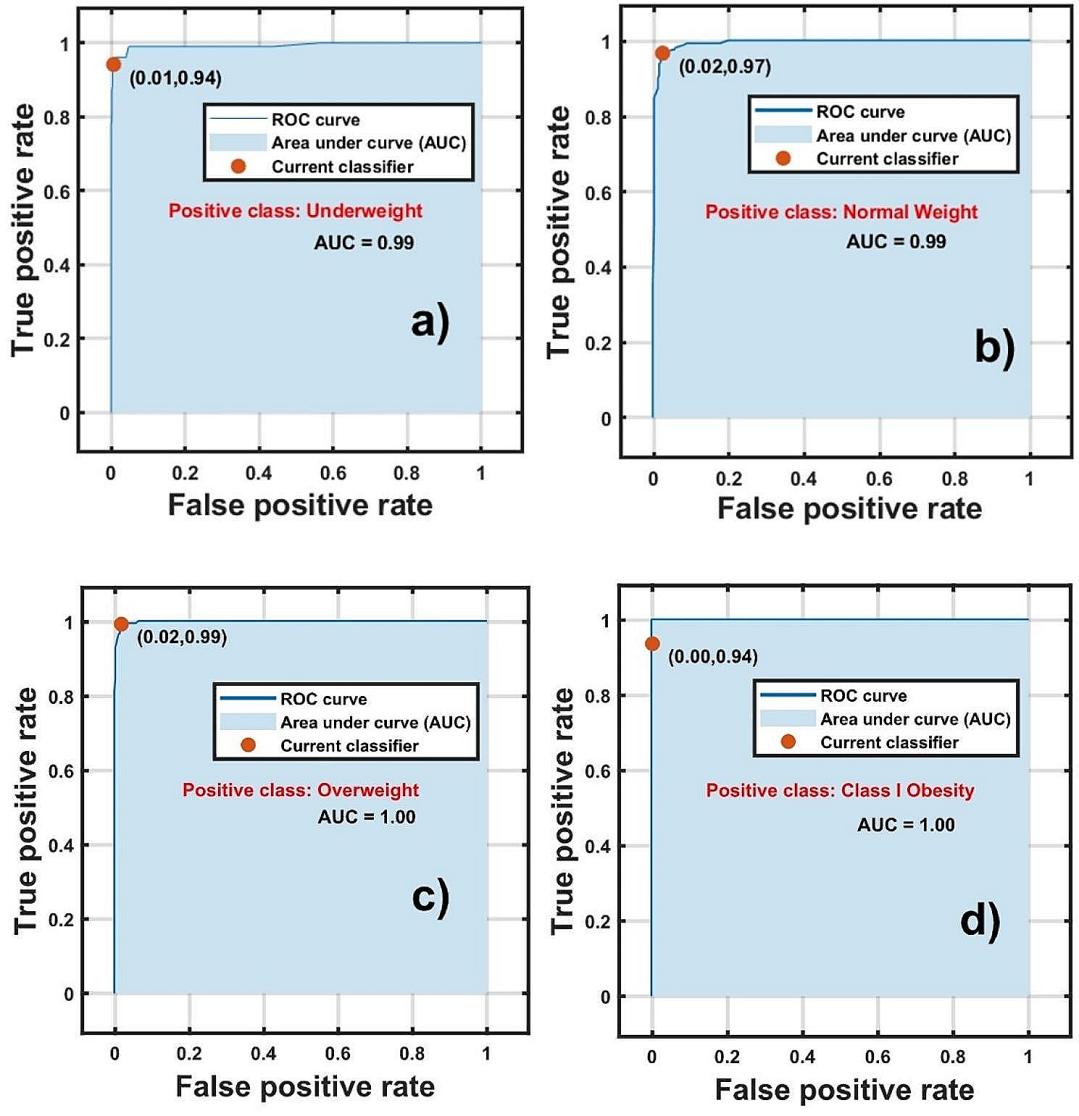
Receiver operating characteristic curve (ROC) for each positive class considered in adolescent cohort

**Fig. 5:**
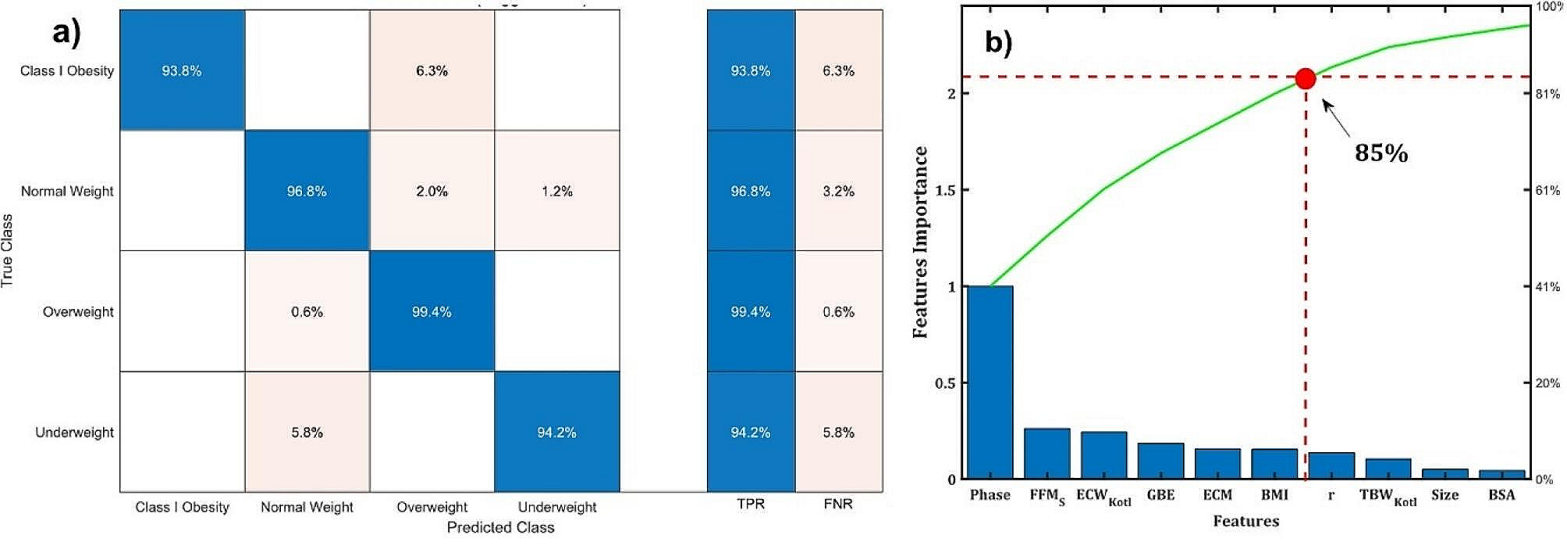
**(a)** Correlation matrix and **(b)** Pareto plots of the weight status classification model of the adolescent cohort

The ROC for each weight class is shown in Fig. 4. The AUC values exceed 0.99, indicating that the classification model has excellent accuracy. Figure 5a illustrates the correlation matrix obtained from the training data. The model achieves a 93.80% accuracy in predicting class I obesity, with a 6.28% false negative rate for overweight assignments. Normal weight status has a 96.80% accuracy, with a 3.19% false negative rate for assignments between overweight and underweight. Overweight predictions exhibit 99.40% accuracy, with a mere 0.61% false negative rate for normal weight assignments, while underweight predictions have a 94.21% accuracy with a 5.82% false negative rate for normal weight assignments.

These false negative rates can be interpreted as transitions between weight statuses. For example, class I obesity is defined by WHO as having BMI between 30.00 and 34.99 Kg/m^2^, while overweight lies between 25.00 and 29.99 Kg/m^2^, and the boundary between these statuses is narrow with a BMI difference of only 0.01 Kg/m^2^ [12, 25]. Therefore, the 6.32% false assignment of overweight to class I obesity can be attributed to a transitional state from overweight to class I obesity. Similar conclusions can be drawn for normal and underweight statuses. This is crucial for early diagnosis and implementing treatment strategies to manage weight status of the adolescent cohort.

Figure 5b displays the feature importance of the adolescent cohort in a Pareto plot. The most influential features, which follow the 85 − 15 Pareto rule, are: Phase, FFM_S_, ECW_Kotl_, basal energetic rate (GBE), total extracellular mass (ECM), and BMI in descendent order. Contrary to reports in the literature stating that impedance measurement is not effective in predicting weight status [34, 35]. Figure 5b shows that the phase angle is a robust parameter for predicting weight status in adolescents.

## Conclusions

In this work, a predictive classification and regression learner model is used to study the association of weight status as a possible risk of chronic kidney disease of healthy infant-juvenile cohort from the Pediatrics Hospital, Santiago de Cuba, Cuba. The present study used 19 characteristics derived from bioimpedance measurements, including other physical parameters. The classification model shows that there are other characteristics different than body mass index that can be used as a predictors of weight status (the size, body density, phase angle, body mass index, fat-free mass (FFM_S_), total body water volume according to Kotler, body surface area, extracellular water according to Kotler and sex). For a specific adolescent cohort, the most influential parameter determining the weight status is the phase angle. The regression learner model was trained with the data and the abovementioned characteristics, predicting with high accuracy the weight status of the volunteers.

## Data Availability

The datasets used during the current study available from the corresponding author on reasonable request.
